# Animals in Respiratory Research

**DOI:** 10.3390/ijms25052903

**Published:** 2024-03-01

**Authors:** Eleonore Fröhlich

**Affiliations:** 1 Center for Medical Research, Medical University of Graz, 8010 Graz, Austria; eleonore.froehlich@medunigraz.at; 2 Research Center Pharmaceutical Engineering GmbH, 8010 Graz, Austria

**Keywords:** respiratory barrier, animal models, chronic respiratory diseases, respiratory tract, mammals, zebrafish, fruit fly, drug development

## Abstract

The respiratory barrier, a thin epithelial barrier that separates the interior of the human body from the environment, is easily damaged by toxicants, and chronic respiratory diseases are common. It also allows the permeation of drugs for topical treatment. Animal experimentation is used to train medical technicians, evaluate toxicants, and develop inhaled formulations. Species differences in the architecture of the respiratory tract explain why some species are better at predicting human toxicity than others. Some species are useful as disease models. This review describes the anatomical differences between the human and mammalian lungs and lists the characteristics of currently used mammalian models for the most relevant chronic respiratory diseases (asthma, chronic obstructive pulmonary disease, cystic fibrosis, pulmonary hypertension, pulmonary fibrosis, and tuberculosis). The generation of animal models is not easy because they do not develop these diseases spontaneously. Mouse models are common, but other species are more appropriate for some diseases. Zebrafish and fruit flies can help study immunological aspects. It is expected that combinations of in silico, in vitro, and in vivo (mammalian and invertebrate) models will be used in the future for drug development.

## 1. Introduction

Humans are exposed to inhaled particles as part of environmental pollution, in the workplace, or as aerosols in medical treatment. Despite significant advances in alternatives to in vivo testing, animal studies are still the gold standard for toxicity testing and drug development. Important parameters for relevant testing are the correct administration of the aerosol and the selection of an appropriate animal species. Generation of the test atmosphere, including its characterization, application by whole-body, mouth-only, and nose-only exposure, and selection of the appropriate animal model are critical steps in the in vivo evaluation of efficacy and toxicity. Inhalation toxicity is regulated by Organisation for Economic Co-operation and Development (OECD) guidelines [1] and preclinical testing of orally inhaled drugs is guided by agencies such as the Food and Drug Administration (FDA), the European Medical Agency (EMA), or the Pharmaceuticals and Medical Devices Agency (PMDA) [2]. Regulatory authorities in Europe and North America require safety studies for approval of first-in-human (Phase I) studies, but there is currently no requirement to provide evidence of potential efficacy [3].

In September 2022, the European Parliament overwhelmingly voted in favor of a resolution to phase out the use of animals in research, testing, and training. Between 2015 and 2018, the average annual percentage decrease in animal use for basic, applied, and translational research in 28 Member States (excluding Norway) was 1.6% [4]. If the current annual decrease of 1.6% is maintained, the use of animals will not be halved even by 2050. Given this, a complete replacement of animal studies does not seem realistic in the near future, and a re-evaluation of other options to reduce animal use seems necessary. This review will describe the contributions of animals in respiratory research, with the aim of highlighting their importance and limitations for each disease. It will begin with a brief history of animal studies and continue by illustrating species-specific differences in the respiratory systems of the mammals most commonly used to study the respiratory system. After summarizing the use of mammals in the major chronic respiratory diseases, the status of non-mammalian models, such as zebrafish and fruit fly, will be mentioned.

## 2. History of Animal Experimentation in Medical Research

The use of animals in medicine has changed over time. The first animal experiments were performed by Alcmacen of Croton in the 6th–5th centuries BC [5]. Aristotle, Erasistratus, and Galen were important figures in the history of animal experimentation in the 4th–2nd centuries BC. At that time, the similarity of humans to other mammals was recognized, and the main goal of experiments was to better understand physiological processes in humans. By the 12th century AD, animals were being used to test surgical procedures, for instance, tracheostomy on goats by Ibn Zuhr [6]. Vivisection was the dominant technique. While in the Middle Ages there was little scientific activity, in the Renaissance comparative anatomy was started by scientists like Vesalius [7]. During the 18th and 19th centuries, vivisection was practiced by Magendie and Bernard. Francois Magendie is regarded as the founder of modern physiology and is notorious for the crudeness of his experiments [8]. Claude Bernard, the student of Magendie, advocated the use of properly controlled and rigorously conducted animal experiments, thereby setting the standard for experimental medicine [9]. In the early and mid-nineteenth century, the availability of general anesthetics and the increasing popularity of domestic pets fueled anti-vivisection sentiments, which culminated in the “Cruelty to Animals Act” of 1876, the first piece of legislation ever to regulate animal experiments [10].

Domesticated rats were the first rodent species used for scientific purposes, and the Wistar rat was developed in 1909. The similarity of mammals to humans was later used for drug testing, as required by the FDA since 1938 [11]. The Federal Food, Drug, and Cosmetic Act of 1938 mandated safety testing of drugs after the tragic incident involving ‘Elixir Sulfanilamide’. The drug contained raspberry flavoring dissolved in diethylene glycol (DEG) to make it more appealing to users, but this ingredient had not been tested in animals. As a result, the product caused the deaths of over one hundred people.

The use of animals also had practical aspects. Humans require 3000 times higher doses of penicillin than mice, and laboratories in 1940 would not have been able to produce enough drug for human testing. Animal experiments were crucial in the development of vaccines, anesthetics, transplantation protocols, insulin, chemotherapeutics, and gene therapies [12]. Guinea pigs, rabbits, and monkeys have been the most important species for asthma drug development. 

Milestones in animal models were the generation of the transgenic mouse in 1980 and the first gene knockout mouse in 1988 [13]. The use of animals increased with this new technique, which allowed the role of specific genes in disease pathology to be assessed. The concept of replacement, reduction, and refinement (3Rs) of animal experimentation, developed by Russell and Burch in 1959, was not immediately accepted by the scientific community.

**Figure 1:**
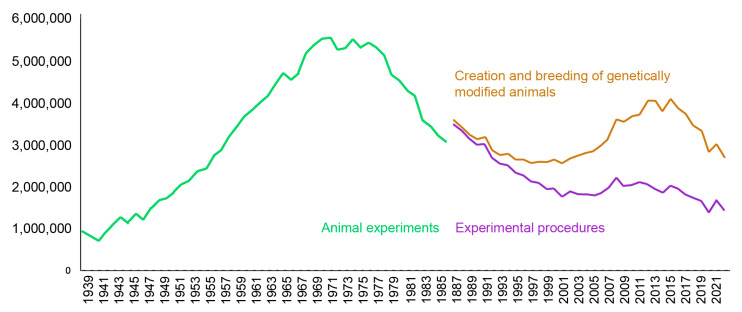
Number of animals used in the UK between 1939 and 2022 (based on information from the yearly reports of the Home office, available at [17]).

The regulatory environment for animal use is variable across countries [14]. In some countries, individual scientists are licensed to conduct animal-based research, while in others, the institution may hold the license. Some countries require an ethical review of proposed research, while in others, no institutional or governmental review of the proposed study is necessary. Appropriate training qualifications of researchers are not mandatory in all countries. Detailed documentation of animal procedures is available for the United Kingdom because every year the Home Office releases the statistics on animal research in Great Britain for the preceding year (the most recent report [15]). It reflects the trend seen in many European countries. Approximately one million animal experiments were performed between 1939 and 1941 (Figure 1). This number increased to more than five million between 1968 and 1978. In 1987, the reporting requirements were changed to “animal procedures,” which included breeding animals, leading to an increase in the number of animals reported [16]. At that time, genetically modified animals began to be used, and the number of animals increased to four million in 2011–2015. Since then, the number of animals has decreased, and in 2022, 2.76 million animals were reported, of which 50% were genetically modified animals. The European Union has set the complete replacement of animal testing in biomedicine as an ultimate goal, as stated in Directive 2010/63/EU of the European Parliament and of the Council of 22 September 2010. Although dramatic improvements in in vitro studies have been achieved and new technologies have been developed (e.g., microfluidics, 3D culture, reconstituted tissues, etc.), many researchers believe that this is not realistic in the near future.

## 3. Differences in the Respiratory Tract of Commonly Used Animals

**Figure 2:**
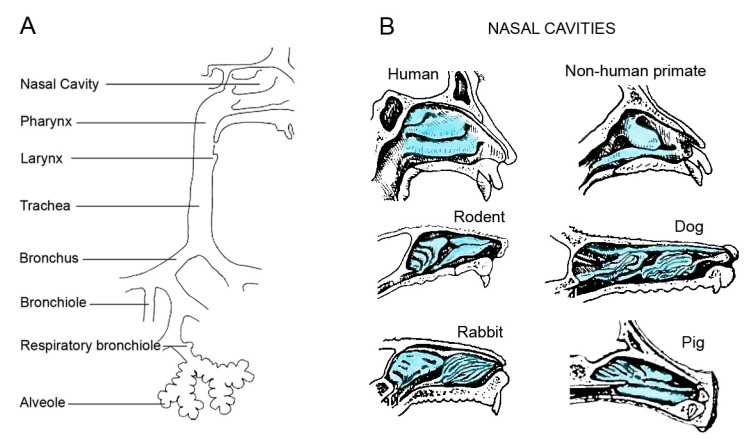
The parts of the human respiratory tract (**A**) are similar to those of other mammals, but there is marked interspecies variation in the size and shape of the nasal turbinates (**B**), shown in light blue).

Not all mammalian species are used in medical research. Cats have historically been very important, but they are currently less important in animal experimentation. In the United Kingdom, for example, only 0.004% of the animals used for scientific purposes in 2022 were cats [18]. Although horses naturally develop equine asthma syndrome, they are not used as animal models in respiratory research. They are mainly used for regulatory purposes, particularly the production of blood-based products for a variety of diagnostic purposes [19]. Cattle are suitable for screening anti-tuberculosis (Tbc) vaccines and drugs, but the species has no role in other chronic lung diseases [20]. This review includes species used in the testing of more than one chronic respiratory disease. The composition of the respiratory tract in the upper and lower airways is similar in mammals [21]. Lung volume, lung surface area, and harmonic barrier thickness are linearly related to body mass. The upper airways consist of the mouth, nose, sinuses, pharynx, and larynx, and the lungs consist of the tracheobronchial region (trachea, bronchi, and terminal bronchioles) and the alveolar region (respiratory bronchioles, alveolar ducts, and alveoli) (Figure 2A). The upper respiratory tract is important for warming and initial clearing of inhaled air; it also has functions in immunology, taste, smell, and speech. The nasal cavity varies in size and shape between species, resulting in different amounts and patterns of particle deposition (Figure 2B). The trachea and bronchi conduct inhaled air to the alveoli, where gas exchange takes place. The conducting airways are important sites for airway protection (mucociliary clearance) and diagnosis (bronchoalveolar lavage), a target for the chronic obstructive airway diseases asthma, chronic obstructive pulmonary disease (COPD), and cystic fibrosis (CF), and a region for transformation (bronchial carcinoma). The airways are less accessible for diagnostic procedures, but they are the target for cell damage (acute lung injury, inflammation) and extracellular matrix alterations (restrictive airway diseases).

## 4. Physiology and Anatomy

Some physiological parameters are related to size, and there is an allometric relationship between minute volume and body weight (BW), and usually smaller species breathe faster than larger species [22]. The respiratory rate (breaths/minute) decreases from mice (272), rats (70–120), guinea pigs (80), rabbits (35–100), ferrets (30–40), dogs (15–30), sheep (16–30), and humans (12–20) to pigs (14–18) [23,24]. For physiologically relevant testing of aerosols, the breathing pattern is also relevant. While humans can breathe through the nose and mouth, rodents, and rabbits are obligate nasal breathers, resulting in higher particle deposition in the nose (Table 1). The cough reflex is important in protecting the lungs from inhaled particles, and coughing is a manifestation of asthma. The absence of this reflex in some species (rodents, rabbits) is a limitation in the evaluation of aerosols and the identification of antitussive drugs.

All mammals have two lungs, which may be divided into lobes. The left lung has fewer lobes than the right lung, but the number of lobes into which the right and left lungs are organized differs between 1 and 3 in the left lung and 3 and 4 in the right lung. The respiratory tract may have two different branching patterns. When child branches are generated at the sidewall of the parent branch, it is called monopodial branching. Bifurcation at the tip of the parent branch is called dichotomous (dipodial) branching. With the exception of sheep, which have an irregular pattern, only humans and non-human primates have dichotomous branching of the bronchi [36,39]. Some mammalian species (e.g., sheep and pigs) have a tracheal bronchus that arises from the trachea, or the right primary bronchus. In humans, it is regarded as an anomaly [40]. The number of branches, called generations, for a given species varies in the literature and has been reported to be greater in some species than in humans. Several orders of respiratory bronchioles are present in humans, dogs, ferrets, and monkeys, located between terminal bronchioles and alveolar ducts/alveoli, resulting in the transition from fully ciliated tracheobronchial airways to fully alveolated alveolar ducts [35]. The presence is not related to size, as sheep have also been reported to have poorly developed respiratory bronchioles [36]. The lungs of rats and mice are essentially devoid of respiratory bronchioles. Their absence appears to result in lower deposition and relatively rapid early alveolar clearance of insoluble particles in rodents, whereas alveolar clearance is much slower in humans, guinea pigs, dogs, and monkeys [35,41]. Other differences concern the composition of the tracheobronchial tree. Cartilage is restricted to the upper third of the initial intrapulmonary airways in mice, whereas the walls of the smaller portions of the upper airways in humans, monkeys, sheep, ferrets, and rats also have cartilage [42,43]. The lack of submucosal glands in rodents and rabbits is a major limitation in the study of obstructive lung diseases, which are usually associated with overproduction and/or changes in mucus composition. The greater compliance of the chest wall in small animals makes them more sensitive to distending pressures [44]. Prominent interlobular septa are seen in sheep and pig but not in the smaller animals [45]. If thick fibrous membranes separate the lobes, different experiments can be performed in one lung, and fewer animals can be used.

Mucociliary clearance may also differ between species, but direct comparison is hampered by different values reported in the literature for the same species. Felicetti et al. studied mucus transport in the trachea of rats, guinea pigs, rabbits, dogs, and humans in vivo and found a linear increase in velocity from rats (1.9 ± 0.7 mm/min) to humans (15.5 ± 1.7 mm/min) [46]. The authors compared their data with velocities measured in other publications (5.1–8.1 mm/min in rats, 7.5–19.2 mm/min in dogs, and 3.6–21.5 mm/min in humans), and explained the differences with the different experimental conditions (material, anesthesia, temperature, and strain). The data obtained in excised tracheal samples were always lower than those obtained in vivo [47]. Mucus transport rates of porcine and ovine tracheas in vivo were 6.9 ± 0.7 mm/min and 8.2 ± 1.9 mm/min, respectively, compared to 1.32 ± 1.2 mm/min and 1.62 ± 1.2 mm/min in vitro. There were no marked differences in ciliary beating frequency between pigs (12 Hz), sheep (13.7 Hz), and rabbits (10 Hz), but transport velocity was higher in excised sheep tracheas than in the other species [48]. Since the trachea and bronchi are longer in larger species than in smaller species, similar mucus transport will result in faster particle clearance in small animals such as rats than in larger species such as humans. Even under the assumption of slower mucus transport (e.g., 1.9 mm/min for rats and 5.1 mm/min for humans), in silico models predict faster particle clearance from the respiratory tract in rats than in humans [49]. While 10–15% of particles initially deposited in the bronchial tree were retained in the human lung after 24h, all particles were cleared from the rat lung after 6–8 h.

Mammalian lungs also differ in the blood supply provided by the bronchial arteries. In general, the lungs receive oxygen-poor arteries (pulmonary arteries) and oxygen-rich vessels (bronchial arteries). In the human lung, the bronchial arteries also supply the periphery of the lung, whereas in small animals such as mice, they are restricted to the trachea and main bronchi [50]. Pulmonary venules are located in interlobular tissue in humans, monkeys, and sheep, but adjacent to bronchioles in mice and rats [43]. These differences can result in different effects of hypoxemia.

The importance of animal models for human translation is often not clear, and investigators’ decisions are often based on cost, availability, and investigator experience. It may be helpful to determine importance by comparing established drugs in a rodent and a larger animal. This approach is required by the FDA for preclinical safety evaluation [51].

## 5. Histology

Differences in the cellular composition of the airway mucosa play a role in disease-specific animal models. Table 2 shows the cellular composition of the respiratory tract until the start of the subsegmental bronchi before the start of bronchiole at generation 12–15 [52]. The thickness of the epithelium is loosely correlated with the size of the species, being thinner in the small species, whereas the number of cells/mm basement membrane appears to be independent of size (Table 2).

**Figure 3:**
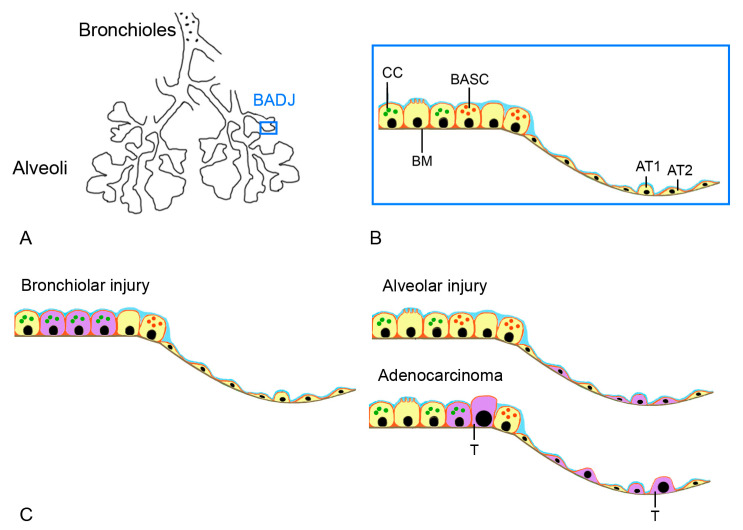
(**A**): Localization of the transition between bronchioles and alveoli, termed broncho-alveolar junction (BADJ) in mice. (**B**): The zone is composed of club cells (CC), bronchoalveolar stem cells (BASC), alveolar type 1 (AT1), and alveolar type 2 (AT2) cells. (**C**): During bronchiolar injury, mainly club cells are replaced, and during alveolar injury, AT1 and AT2 cells are replaced (replaced cells are indicated by purple color). Transformation results in the formation of new bronchiolar, alveolar, and tumor cells (T). Abbreviations: BM, basal membrane.

The percentage of ciliated cells in the epithelium of the conducting airways (trachea and bronchi) is similar to the human situation in all mammalian models (32–49% of all cells). However, intraepithelial secretory cells, goblet cells, serous cells, and club cells (formerly Clara cells) show species-specific differences. Only humans, guinea pigs, ferrets, monkeys, dogs, and sheep have significant amounts of goblet cells [32,53,55,57,58]. Serous cells are found only in mice [54] and club cells in mice and rabbits [54,56]. In larger mammals, club cells are absent in the proximal airways. However, they represent a prominent population in the human terminal (11 ± 3%) and respiratory bronchioles (22 ± 5%) [59]. Unlike rabbits and mice, club cells were not found in the trachea and major bronchi of guinea pigs [60]. The percentage of basal cells, which are responsible for cell regeneration, shows marked variation between species, which can be explained by the fact that, depending on the species, club cells are also involved in the regeneration of the epithelium of the conducting airways [61]. The architecture of the zone between the bronchiole and the alveoli shows species-specific differences (Figure 3A).

In the mouse, the airways open into the alveoli at the bronchioalveolar duct junctions (BADJs), which consist of club cells and bronchial epithelial cells [62]. This zone is important because it contains the bronchoalveolar stem cells (BASCs), which contribute to the regeneration of both the distal airways and the alveoli and provide bronchiolar club cells, ciliated cells, goblet cells, and type I and type II alveolar epithelial cells [54] (Figure 3B). In humans, the zone is less well defined and contains the respiratory bronchioles with multiple alveolar ducts. Damage to this zone impairs the ability to regenerate and induces the alteration and transformation of lung cells. In COPD, cigarette smoke (CS) leads to stem cell exhaustion, defective differentiation, and reduced capacity to generate normal lung epithelia even after CS exposure ends [63]. BASCs are involved in bronchial cell hyperplasia, squamous metaplasia, goblet cell hyperplasia, reduced ciliary length, and reduced barrier function. Somatic mutations induced by CS can result in cell transformation. In COPD and CF, BASCs act as a combination of bronchiolar and alveolar injuries (Figure 3C). In idiopathic pulmonary fibrosis (IPF), the role of AT2 cells has been demonstrated [64]. The proximal alveolar acinus is susceptible to air pollutants, and BASCs may give rise to the transformed (club and alveolar) cells of lung adenocarcinoma. Accelerated aging of AT2 cells, which represent resident alveolar stem cells, is thought to contribute to emphysema in human patients (e.g., COPD) [65]. Stem cells are also found in the trachea and main bronchi, basal cells of the respiratory epithelium in humans, basal cells in the trachea, and club cells and secretory cells in rodent bronchi [50].

## 6. Use of Healthy Animals

Healthy animals are used for training in surgical procedures and for toxicity testing. For both uses, treatment protocols and outcome parameters are well characterized [66]. Pigs are used for training in surgical procedures, such as thoracoscopic lung segmentectomy, or for routine procedures, such as tracheostomy, thoracotomy, vascular treatment, thoracotomy, and chest tube placement. The treatment is identical to that in humans, and the similar organ size to humans is advantageous [67,68]. After primates, pigs have the most similar airway morphology and epithelial composition to humans among other larger animals [69].

Healthy rats are mainly used for toxicity testing of inhaled vapors and particles according to established guidelines. For hazard identification and screening, animals may be dosed with test chemicals by intratracheal instillation or aspiration. Although instillation or aspiration exposure is a simple and inexpensive way to study test chemicals, it is not a substitute for inhalation toxicity studies because the particle distribution does not reflect normal breathing and mucociliary clearance. For single and repeated dose inhalation toxicity testing, non-invasive administration, such as whole body or nose-only exposure, of the aerosols should be used [1,70]. Outcome parameters should include food and water consumption, body weight, lung morphology, respiratory reflexes, lung function, bronchoalveolar lavage (LDH activity, total protein or albumin levels, cell counts and differential for alveolar macrophages, lymphocytes, and neutrophils), and lung burden. For subchronic toxicity studies, food and water consumption, body weight, hematology, clinical chemistry, bronchoalveolar lavage analysis (see above), lung burden, ophthalmologic examination, gross pathology and organ weights, and lung function should be reported [1].

## 7. Respiratory Diseases

The European Union has a high burden of chronic diseases: asthma, respiratory allergies, COPD, occupational lung diseases, sleep apnea syndrome, pulmonary hypertension (PH), lung cancer, and specific communicable lung diseases, especially Tbc [71]. Besides the classification of respiratory diseases into acute and chronic, the classifications based on the affected part of the lung with (i) airways, (ii) alveoli, (iii) interstitium, (iv) blood vessels, (v) pleura, and (vi) chest wall can be used. Representatives (chronic diseases are in bold) of (i) are **asthma**, **chronic obstructive pulmonary disease**, **bronchitis**, **cystic fibrosis**, (ii) pneumonia, **tuberculosis**, pulmonary edema, **emphysema**, **lung cancer**, acute respiratory distress syndrome, **asbestosis**, (iii) **idiopathic pulmonary fibrosis**, **sarcoidosis**, (iv) **pulmonary hypertension**, pulmonary embolism, (v) pleural effusion, pneumothorax, **mesothelioma**, and (vi) obesity, **hypoventilation syndrome** [72].

### Prevalence

Cardiovascular disease is one of the most common chronic diseases. The prevalence is highest in Europe (11,646 cases/100,000) and lowest in Korea (4518 cases per 100,000 people) [73]. Asthma is the most common respiratory disease, but with a global prevalence of 3416 cases per 100,000 [74] in 2019, it is less common than cardiovascular disease. There are also more than 84 rare respiratory diseases [75]. According to the World Health Organization, a rare disease is a health condition with a low prevalence compared to common diseases, affecting approximately 6% of the world’s population [76]. European Union (EU) countries define rare diseases as “life-threatening or chronically debilitating diseases with such a low prevalence (less than 5 per 10,000) [77]. In the United States of America (USA), “rare disease” means any disease or condition that affects fewer than 200,000 people in the USA, or up to 7 per 10,000 [78]. In China, Australia, and Japan, a rare disease affects up to 1 per 10,000, 1.1 per 10,000, and 4 per 10,000 people, respectively [78,79].

The fact that they belong to the group of rare diseases does not mean that they are not relevant for animal studies. A PubMed search (“cardiovascular disease”[title/abstract] AND (“mouse”[title/abstract] OR “rat”[title/abstract] OR “guinea pig”[title/abstract] OR “rabbit”[title/abstract] OR “dog”[title/abstract] OR “sheep”[title/abstract] OR “ferret”[title/abstract] OR “pig”[title/abstract] OR “non-human primate”[title/abstract])) AND (2003/1/1:3000/12/12[pdat]) retrieved 4456 articles (search on 19 October 2023). Mouse and rat studies accounted for 93%. The same search using “chronic obstructive pulmonary disease,” “cystic fibrosis,” “pulmonary hypertension,” “idiopathic pulmonary fibrosis,” and “tuberculosis” instead of “cardiovascular disease” identified between 920 (IPF) and 2976 (Tbc) studies. Between 77% (tuberculosis) and 98% (idiopathic pulmonary fibrosis) of the studies were conducted in rodents.

## 8. Disease Models

Animal models for the study of respiratory diseases are designed to mimic changes in the human lung as closely as possible. The exception is lung cancer, where xenografts, in which human tumor cells are injected into the flank of rodents, are common and used in 97% of studies. For the drug screening of chronic respiratory diseases, the presence of key disease symptoms is important. Animal models based on phenotype characterization were identified through PubMed, Science Direct, and Google (Scholar) searches.

There are two ways to produce symptoms similar to human disease in animals: induced models and genetically modified animals. Healthy animals exposed to certain drugs or procedures are called “induced,” while knockout and transgenic animals belong to the latter group. The ability to generate genetically modified animals has opened up new possibilities, but it also has its limitations [80]. Genetic modifications can be global if embryonic stem cells (knockout animal) or oocytes (transgenic animal) are used. Tissue-specific expression requires tissue-specific promoters. Conditional tissue-specific expression requires tissue-specific promoters plus a reverse tetracycline-controlled transactivator (rtTA) combined with a tetracycline response element (TRE). Exposure to doxycycline results in tissue-specific gene expression. The Clustered Regularly Interspaced Short Palindromic Repeats (CRISPR)/Cas9 technology is currently the most often used method for genome editing [81].

The success of drug screening in chronic respiratory diseases is variable and particularly low for IPF, where screening has shown anti-fibrotic effects of >500 compounds and none has been translated into a therapeutic agent [82]. This is partly due to the experimental setting. Drugs are usually given as prophylactic treatment, and effects on inflammation and early fibrosis are observed in animal studies, whereas in humans, the drugs must act when there is little inflammation and prominent fibrosis. Other important factors may be the use of young mice and the use of bleomycin as a fibrosis inducer. Young mice, similar to humans, are more resistant to fibrosis than aged mice. Further, the bleomycin model does not produce all relevant changes in the mouse lung. The mouse bleomycin model is also a good example of the numerous influences in animal models, such as strain, age, sex, and route of administration [83,84]. Differences in species-specific cellular responses between mice and humans may explain translation problems in asthma [85]. Plasma exudation with leukocyte infiltration and growth factor-active proteins characterizes human asthma, and the extent of exudation and eosinophil degranulation correlates with disease severity. Although eosinophils also accumulate in mice, they do not degranulate [86]. In addition, rodent mast cells release serotonin, which has no established role in human asthma [87]. Similar problems apply, to varying degrees, to other respiratory diseases and species.

The species are grouped into rodents (mouse, rat, guinea pig), other small laboratory animals (ferret, rabbit), and larger species (non-human primates, dogs, sheep, and pigs) for the different diseases. Guinea pigs are traditionally classified as rodents, but sequence analysis of the complete mitochondrial genome suggests that they do not belong to the myomorph rodents [88]. Another group, who re-analyzed the data, concluded that the proofs that guinea pigs were not rodents resulted from systematic error associated with an oversimplified model of sequence evolution [89]. In this review, guinea pigs will be regarded as rodents.

### 8.1. Asthma

The clinical manifestations of human asthma are coughing, wheezing, chest tightness, and shortness of breath [90]. Increased airway hyperresponsiveness (AHR) to methacholine and decreased forced expiratory volume in 1 s (FEV1) are important diagnostic parameters. Asthma used to be considered a single entity, but this concept is no longer valid as different endotypes and different phenotypes have been identified. The main differentiation in the current classification is into T2 and non-Th2 asthma [61]. The T2 endotype has the phenotypes atopic, late onset, and aspirin-exacerbated respiratory disease, while the non-T2 endotype is classified into non-atopic, smoker, obesity-related, and elderly phenotypes. Histologic features of asthma include inflammatory cell infiltration of the bronchial mucosa, epithelial cell desquamation, goblet cell hyperplasia, and submucosal thickening. Local overproduction of T2 cytokines (interleukin (IL)-4, IL-5, IL-9, and IL-13) by T2 cells plays an important role in its pathophysiology [91]. Neutrophils, lymphocytes, and macrophages are increased in bronchoalveolar lavage (BAL) fluid. In non-T2 asthma, IL-8 levels and neutrophils are increased.

Asthma symptoms in animal models can be produced in mice, rats, guinea pigs, rabbits, ferrets, dogs, sheep, and non-human primates. Ovalbumin (OVA), house dust mite (HDM), fungal elements (*Alternaria alternata*), pollen, and cockroach extracts can be used as inducers, and intraperitoneal or subcutaneous injection, intratracheal instillation, or aerosol can be used as routes of administration.

#### 8.1.1. Rodents

Acute exposure of mice to OVA produces some features of T2 asthma, but no robust lung function response equivalent to FEV1 changes is seen [56]. The choice of strain is very important. BALB/c or C57BL/6 mice are the most commonly used mouse strains. While BALB/c mice are IgE high responders/Th2 immune responders, C57BL/6 mice show a more Th1 immune response and are low IgE producers. Murine non-T2 asthma models have been generated, but most rely on adoptive transfer of specifically differentiated T cells. The inflammation is short-lived, making longitudinal studies of eosinophilic or neutrophilic airway inflammation in the mouse impossible [92]. Differences in mechanistic processes between mice and humans mean that drug responses in acute OVA-induced asthma in mice are not predictive of efficacy in human asthma [93]. Among the differences is that the mice develop allergic alveolitis-like inflammation rather than typical asthma. In addition, the role of eosinophils and mast cells differs between humans and mice. Histamine and serotonin released by mast cells are involved in mouse AHR, whereas the role of serotonin in human AHR is unclear [94]. Chronic asthma is difficult to mimic in mouse models because the animals develop tolerance to the allergen, which can be prevented by repeated administration of OVA or HDM. Limitations include the lack of β2 adrenergic receptors on smooth muscle and the cough reflex, different lung anatomy, and marked strain differences [93]. The low mucus production due to the lack of submucosal glands and fewer goblet cells limits the similarity between human and mouse asthma.

Despite the differences in cytokine release and cellular composition of the lung infiltrate, mice play a prominent role in the identification of the roles of specific cytokines [95]. Specific overexpression of IL-4, IL-5, IL-13, IL-9, IL-11, and vascular endothelial growth factor (VEGF) is achieved by using a promoter for a gene expressed predominantly/exclusively in the lung [96]. Deletion of Th2 locus control region (LCR), metalloproteinase (MMP) 9, and MMP-8 can limit airway inflammation [97]. Some features of asthma, such as AHR, tissue remodeling, and eosinophilia, were induced in all transgenic models. Targeted deletion of the T1 transcription factor T-bet resulted in increased TGF-β and a phenotype similar to the cytokine overexpression models. Because of fetal lethality, only conditional expression of transforming growth factor (TGF)-β is possible. Although these models may provide more insight into the regulation of asthma, the extreme overexpression most likely overemphasizes the role of each molecule. Furthermore, the lack of allergen challenges in these models makes it impossible to assess the immunological pathways that induce chronic inflammation.

The exacerbation of all obstructive lung diseases is caused by infections. In mice, these exacerbations can be produced by HDM sensitization followed by infection with influenza virus, respiratory syncytial virus (RSV), human rhinovirus (HRV), or polyinosinic:polycytidylic acid (polyIC) stimulation [93]. Virus-induced asthma models caused more pronounced increases in AHR when mice were challenged with HDM than with OVA [98]. The responses to influenza virus and RSV were more robust than those for HRV. The duration of the sensitization with HDM varied from two to seven weeks [99,100].

Acute OVA exposure in rats shows variable airway responses and reproduces some features of human asthma, namely AHR, Th2 phenotype, and an increase in eosinophils [93]. Some rat strains, notably Brown Norway rats, show a weak bronchoconstrictor response. Intravenous injection and the use of adjuvant is needed for sensitization and tolerance against the antigen is common [101]. The lack of robust changes in lung function and inter-laboratory variability, as well as the paucity of important cell types (goblet cells) in this disease, are major limitations.

The guinea pig is widely used as an asthma model. The guinea pig has the most prominent smooth muscle compared to the mouse, rat, rabbit, monkey, dog, sheep, and pig, which makes this species an ideal model for the evaluation of bronchiodilators [102]. The most commonly used allergen to induce asthma is OVA, although HDM is also possible. Guinea pigs have the advantage that there are several similarities to human asthma: histamine H1 and the leukotriene cysteinyl leukotriene (cysLT)-1 receptor are involved in mediating airway smooth muscle contraction, and eosinophil infiltration, AHR, and mucus production are similar [94]. Acute OVA exposure in guinea pigs produces responses similar to human FEV1, but responses vary between laboratories [93]. Guinea pigs become tolerant to allergens like mice, so there are no chronic asthma models [56]. Despite some similarities to human asthma, there are also differences. Bronchoconstriction in guinea pig asthma models appears to be mediated primarily by histamine, which may have limited translational relevance to human asthma patients. IgG1, not IgE, is the major anaphylactic antibody [103]. Although there are numerous alveolar macrophages in the alveoli, phagocytosis in the guinea pig lung is performed by neutrophils extravasated from blood vessels [94]. Experiments with guinea pigs present the problem of difficult blood collection because they have thick skin and lack a tail. The soft palate makes intratracheal procedures difficult. The limitations to the use of guinea pigs are the long duration of the model and the considerable interlaboratory variability. Exposure of guinea pigs to the parainfluenza virus after sensitization with OVA for three weeks produces a pattern of inflammation similar to human asthma exacerbations [104].

#### 8.1.2. Other Small Laboratory Animals

Historically, the rabbit was the first asthma model. The protocol requires sensitization within 24 h of birth to obtain the late-phase airway response [56]. The inflammatory response in rabbits is characterized by the infiltration of a combination of neutrophils and eosinophils (heterophils), similar to the human lung. Rabbits have been used with good success for drug testing in early and late response, AHR, and eosinophil infiltration [105]. Changes in mucus composition and viscosity in asthma can be less well studied because the mucus composition of rabbits differs from human mucus [106].

Repeated administration of OVA to ferrets increases mucus production [107]. The possibility of studying of mucus changes may make ferrets an interesting model for asthma, as these changes cannot be observed in rodents and rabbits. However, the lack of lung inflammation severely limits the use of ferrets as an asthma model.

#### 8.1.3. Larger Animals

Hypersensitivity in dogs manifests as atopic dermatitis or gastrointestinal symptoms, but they can be sensitized with *Ascaris suum* larvae and ragweed, e.g., [108,109]. The antigen-induced hyperresponsiveness is thought to involve both neutrophils and eosinophils [110]. Dogs have the unique ability to develop prolonged airway hypersensitivity, up to 5 months, to *A. suum* [111].

Sheep and non-human primates are representative models of asthma. In sheep, HDM and *A. suum* extract are used to induce Th2 asthma, and in non-human primates, *A. suum* extract, HDM, and birch pollen allergens are used [36,112,113,114]. Acute and chronic asthmatic changes can be observed. The bronchi of sheep are heavily muscled, but unlike those of the guinea pig, the muscle is less prominent in the distal airways [102]. Compared to humans, sheep present more pulmonary intravascular macrophages.

Pigs are not a good asthma model because of their decreased sensitivity to the allergen after repeated administration [115]. Specific protocols, e.g., *A. suum* extract in Al(OH)_3_, followed by two booster doses and a challenge with a nebulized allergen, must be used to induce eosinophil and neutrophil infiltration resembling human asthma.

Although early- and late-phase bronchoconstriction can be produced in many species (rat, guinea pig, ferret, non-human primate, dog, sheep, and pig), AHR is absent in guinea pig and pig. Mice do not show bronchoconstriction but have AHR. Inflammatory cell infiltration of the lungs is seen in all species except the ferret [87]. The generation of chronic asthma is a problem in small animal models. Exacerbations can be well studied in rodents and guinea pigs.

### 8.2. Chronic Obstructive Pulmonary Disease (COPD)

COPD is an umbrella term for several respiratory diseases, including emphysema, chronic bronchitis, and, in some cases, asthma. COPD, like asthma, is characterized by variable airflow obstruction in combination with AHR. However, unlike asthma, the airflow obstruction is only partially reversible or irreversible, and mucus metaplasia of chronic bronchitis and alveolar destruction cause progressive loss of lung function [116]. Air pollution and cigarette smoke (CS) are the most common causes of COPD in humans.

#### 8.2.1. Rodents

There are several inducers of COPD (CS, elastase, papain, LPS, bleomycin, FITC, silica, etc.) [106]. CS is the most commonly used and best-characterized inducer of COPD, but methods of smoke production, length, frequency, number, and type of exposures (whole body or nose/mouth only) vary and influence the development of emphysema. Small airway modeling is less often induced than emphysema and inflammation [117]. Female A/J mice develop emphysema earlier than male A/J mice [106]. CS produces pulmonary symptoms with an increase in neutrophils and mononuclear cells, systemic inflammation, and weight loss. A major limitation is that lung damage stops when smoke exposure is stopped [118].

Transgenic models are used to gain more information about the mechanisms by which inflammation and emphysema develop. Knockout of MMP-1, MMP12, neutrophil elastase (NE), IL-18Ra, TNFR-α, microtubule associated protein 1 light chain 3 beta (LC3B), nuclear factor erythroid-2-related factor 2 (Nrf2), and overexpression of CuZn superoxide dismutase can protect against CS-induced emphysema and inflammation [97]. Overexpression of IL-13 resulted in inflammation, lung destruction, airway remodeling, goblet cell hypertrophy, and subepithelial collagen deposition, as seen in human COPD, while overexpression of interferon (IFN)-γ resulted in emphysema [119]. Targeted loss-of-function mutations of elastin, fibulin-5, platelet-derived growth factor (PDGF), fibroblast growth factor receptor, surfactant protein D, tissue inhibitor of metalloproteinases-3, ATP-binding cassette 3, and microfibrillar-associated protein 4 were used to induce pulmonary emphysema but also affected other organs [120]. Overexpression of several other genes resulted in early death, precluding their use as a disease model. Tight skin (Tsk), pallid, beige, and blothy C57Bl/6 mice develop COPD but also multisystemic abnormalities [121,122]. The C57BL/6JJcl substrain Mayumi emphysema mouse was reported to have only pulmonary emphysema and no other organ pathologies [122]. The lack of general availability of this model hinders its widespread use. The general limitations, lack of goblet cells, and few mucosal glands in the mouse airway also apply to induced and transgenic mouse models. Bacterial compounds (lipopolysaccharide, LPS) are most commonly used to induce COPD exacerbations. LPS induces lung infiltration with neutrophils and macrophages in mice, followed by alveolar destruction after prolonged treatment [93]. Exacerbation of COPD by acute (two days–two weeks) CS exposure + PolyIC is not widely used because the effects of life viruses are not reproduced [99,123]. Exaggerated inflammation has some features of COPD exacerbation, but no chronicity has been reported.

Rats are less commonly used as COPD models, although they are easier to handle and analyze because they are larger than mice. In addition to CS, airborne (nano)particles can be used to induce the disease [106]. Instillation of ultrafine silica particles, coal dust, or diesel exhaust particles generates oxidative stress and also promotes tumorigenesis. However, the sensitivity to particles is markedly different from that in humans, in part due to the greater mucociliary clearance in the rat lung. Elastase is used to produce emphysema in Wistar rats [120]. Similar to mice, rats lack goblet cells and have few mucosal glands, and mucus effects in COPD cannot be mimicked well.

Guinea pigs develop many of the symptoms of human COPD, including emphysema, acute neutrophilia, increased epithelial permeability, and sustained monocyte recruitment after administration of CS or LPS [124]. Smoke and polyIC administration result in inflammatory responses similar to COPD exacerbations [93]. The main limitation of the guinea pig model is the axon reflex, which is of minor importance in the human lung. The axon reflex is mediated by peptides released from small-diameter, slow-conducting C-fibers and causes bronchoconstriction [102].

#### 8.2.2. Other Small Laboratory Animals

Rabbit models do not mimic human COPD well [106]. A particular disadvantage of the rabbit as a COPD model is its susceptibility to bacterial and viral infections during non-sterile procedures.

#### 8.2.3. Larger Animals

Larger animals are suitable for assessing specific symptoms of COPD, but the understanding of the mechanism is poor compared to rodents [93]. The advantage of dogs as a COPD model is their ability to cough, which is absent in rodents. Mucus production, collagen deposition, and bronchoconstriction are very similar to humans. Interpretation of results is limited by animal heterogeneity and their use due to regulatory issues. Non-human primates are good models to study the effect of maternal smoking and the carcinogenicity of smoking, but high costs and ethical issues limit their use. Sheep are very good models for evaluating smoke-induced changes in mucociliary clearance. They also develop bronchitis upon smoke exposure [125] and emphysema upon protease instillation [126]. A porcine model of COPD is still under development [115].

### 8.3. Cystic Fibrosis (CF)

CF is a genetic disorder caused by mutations in the gene encoding the epithelial chloride channel of the cystic fibrosis transmembrane conductance regulator (CFTR). The defect causes dysfunction of the lungs, gastrointestinal tract, pancreas, liver, and reproductive tract [127]. Hyperabsorption of sodium ions leads to dehydration, mucus accumulation, impaired mucociliary clearance, and bacterial infections. Coordinated Cl^−^ secretion by CFTR and other Cl^−^ channels and Na+ absorption by the epithelial sodium channel (ENaC) are essential for proper volume regulation of airway surface fluid [128]. Ongoing inflammation and infection in CF patients is likely to cause ongoing proteolytic cleavage and activation of ENaC in the airways, leading to hyperactivated ENaC [129]. In addition to modulating the function of mutant CFTR, inhibition of ENaC has been proposed for the treatment of CF. Mutations of the *CFTR* gene have been used to cause CF in mice, rats, rabbits, ferrets, sheep, and pigs.

#### 8.3.1. Rodents

A number of mouse models with mutant CFTR have been published. The mice are usually of C57Bl/6 background, and only congenic KO mice develop the lung phenotype of human CF. Most models carry the mutation in exon 10 of the *CFTR* gene, but exons 1, 2, 3, or 11 can also be mutated [130]. Another possibility that causes the CF phenotype is the overexpression of ENaC. Spontaneous lung infections do not occur in all mouse models, but ENaC mice show impaired pathogen clearance. Mice expressing human mutant *CFTR* (humanized mice) show some promise but are still under phenotypic characterization [131]. Intestinal manifestations dominate compared to pulmonary symptoms [132]. The main problems of murine CF models are the sterile environment, which does not reflect the human situation, strain differences, residual CFTR protein expression, short lifespan of mice, presence of non-CFTR calcium-activated Cl^−^ channels, and differences to human lung architecture [133].

The *CFTR*^−/−^ rat model shares the problem of early postnatal death due to meconium ileus. Although spontaneous lung infections are not seen, the model looks promising for long-term studies of CF disease manifestations [132]. Rats expressing a humanized version of *CFTR* and harboring the ivacaftor-sensitive variant G551D did not show increased ENaC activity, spontaneous lung infection, or mucus plugging, but were suitable to demonstrate the beneficial effects of the CFTR modulator ivacaftor [134].

#### 8.3.2. Other Small Laboratory Animals

CRISPR/Cas9-mediated disruption of the *CFTR* gene was used to generate CF rabbits that exhibit the bioelectric properties of human CF nasal and tracheal epithelia [135]. Other findings in the lungs of CF patients were lacking, leaving rabbits without established CF models.

*CFTR*^−/−^ ferrets express severe lung pathology and lack bacterial clearance [136]. The BAL fluid of KO animals contains various bacterial species, increased levels of cytokines, lactoferrin, and lysozyme, and decreased levels of α1 antitrypsin and apolipoprotein A1 [137]. They develop human-like lung disease and CFTR-dependent secretion in the trachea. Some characteristics, such as the transepithelial potential difference, were not altered between CF and non-CF ferret tracheas. Ferrets showed a high degree of pulmonary manifestations of CF [132].

#### 8.3.3. Larger Animals

There are no non-human primate models of CF, and given the likelihood of early death from meconium ileus as seen in ferrets, sheep, and pigs, the high cost, difficulty of breeding large numbers, ethical concerns, etc., it is unlikely that such a model would provide enough benefit to justify its creation [132].

Sheep carrying the most common F508del mutation and the rarer G542X mutation were developed using the CRISPR/Cas9 technique [138]. The lungs were grossly and microscopically unremarkable at birth, indicating low lung manifestation, and the short survival after birth (24–48 h) prevented further studies.

The lungs of *CFTR*^−/−^ piglets do not show inflammation at birth but have a similar susceptibility to infection with Staphylococcus aureus as children with CF [139]. The cause of the reduced bacterial clearance in the knockout animals was defective macrophage function [140]. It is hypothesized that tracheal abnormalities caused by CFTR loss contribute to the development of CF later in life. Hoegger et al. conclude that impaired mucus detachment after secretion from submucosal glands may be an early defect in CF [141]. The lung manifestation of CF was high in *CFTR*^−/−^ pigs, but the early death of the animals due to meconium ileus limits their use as a CF model [132].

### 8.4. Pulmonary Hypertension (PH)

PH is defined as a disorder with a mean pulmonary artery pressure greater than 20 mmHg at rest [142]. The pathology may be caused by pulmonary artery obstruction (PAH, group 1), left-sided heart disease (group 2), or secondary to chronic lung disease and/or alveolar hypoxia (group 3). In addition, chronic thromboembolic disease (group 4) and unclear or multifactorial mechanisms may cause PH. The most common form of PH in adults was group 2 alone (34.2%) or combined with group 3 PH (29.3%) [143]. Clinical symptoms of PH are shortness of breath, chest pain, and lightheadedness. Pathological findings include vascular remodeling by accumulation of pulmonary artery smooth muscle cells, endothelial cells, fibroblasts, myofibroblasts, and pericytes in combination with perivascular inflammatory cells [144].

#### 8.4.1. Rodents

Exposure to 10–12% oxygen (hypoxia) for 3–5 weeks is a reproducible method to increase right ventricular systolic pressure, followed by remodeling of the pulmonary vasculature in mice [145]. When combined with the VEGF receptor antagonist Sugen 5416, the hypoxia treatment is more effective. Vascular remodeling decreases over 10 weeks when mice are returned to normoxia. *Schistosoma mansoni* infection causes schistosome-related PH in mice by an unknown mechanism. Since other respiratory diseases also cause vascular remodeling, exposure to cigarette smoke (in COPD) and bleomycin (in IPF) can also be used to induce PH [146]. Surgical procedures such as left pneumonectomy or pulmonary artery binding also increase right ventricular pressure [145].

Mutations in activin receptor-like kinase 1 (ALK1), endoglin (ENG), caveolin-1 (CAV-1), and SMAD8, all identified in PH patients, induce PH in mice [147]. Mice lacking mitochondrial uncoupling protein 2 (UCP2) or sirtuin 3 (SIRT3, a mitochondrial deacetylase) spontaneously develop PH [148,149]. Knockout mutations of bone morphogenetic protein receptor type 2, inhibitor of differentiation-1, 5-hydroxytryptamine transporter, tryptophan hydroxylase-1, elastin, peroxisome proliferator-activated receptor, Raf-1 kinase inhibitor protein, apelin, P53, adenosine A2 receptor, and Notch increase right ventricular systolic pressure in mice and muscular hypertrophy in pulmonary vessels in normoxia or hypoxia [150]. Overexpression of calcium binding protein (S100A4/Mts1), of pro-inflammatory cytokines such as IL-6, IL-8, and TNF-α, of OX40L, or gain-of-function mutations in the *HIF2a* gene cause a similar effect [147,151]. Knockout of 5-lipoxygenase activating protein and overexpression of prostacyclin synthase and prostacyclin receptor reduced hypoxia-induced effects, whereas knockout of COX2 and microtubule-associated protein 1 light chain 3B or overexpression of IL-6 increased them [97]. The genetically engineered mouse lines are reviewed in detail elsewhere [152].

Injection of monocrotaline, either sc or ip, is the routine procedure to induce pathology in rats, which is also the species most often used in PH research [151]. Unlike mice, rats are capable of converting monocrotaline to the active metabolite. Injection causes endothelial cell dysfunction, leading to medial hypertrophy and adventitial fibrosis [153]. The severe form of PH develops only when treatment is combined with chronic hypoxia or left lung resection. Monocrotaline-induced PH shows more evidence of parenchymal lung involvement, more perivascular inflammation, and an absence of microangiopathy compared to human PH. An obvious limitation of the use of monocrotaline is its broad toxicity, resulting in numerous extrapulmonary effects, including hepatic veno-occlusive disease, renal insufficiency, and biventricular myocarditis [154]. Despite similar hemodynamic and histologic findings, Fisher rats are more susceptible to heart failure and death than Sprague-Dawley rats [155]. Rat models of PH are further generated by a single mitomycin C ip injection [145]. Monocrotaline-induced PH is a common model for drug screening but has shown little translational value [156]. It has been suggested that when the monocrotaline model is combined with induction by administration of Sugen 5416 in combination with three weeks of hypoxia [157], the identified compounds are more likely to be effective in the clinic. Mutation of the outward rectifier K+-channel in rats can be used to mimic the disease. In general, models induced by multiple factors (e.g., monocrotaline + pneumonectomy or Sugen 5416 + pneumonectomy) reproduce the human pathology better than single-factor induction [145].

Acute and chronic hypoxia can be used to produce PH in guinea pigs [158]. Medial thickness of alveolar duct and terminal bronchiole arteries increased with hypoxia, but there was no significant difference with respect to PIO_2_ = 90, 78, or 65 Torr and duration (10 or 21 days) of hypoxia.

#### 8.4.2. Other Small Laboratory Animals

Rabbits are not a standardized model for PH, although increases in right ventricular systolic pressure and thickening of pulmonary vessels can be induced by injection of mitomycin C or platelet activating factor [159,160].

#### 8.4.3. Larger Animals

Monocrotaline is also an inducer of PH in dogs. It must be administered as monocrotaline pyrrole, probably because the adult dog liver lacks the enzymatic components necessary to convert monocrotaline to its active metabolite [161]. A single intraperitoneal injection of monocrotaline also leads to PH in mini-pigs [162]. In contrast to small animals, right ventricular hypertrophy can be monitored using non-invasive techniques, which is a great advantage in reducing the number of animals. Injection of Sephadex beads into the pulmonary circulation induces PH in sheep and may be a good model to study the development of right ventricular hypertrophy in chronic PH [163].

### 8.5. Idiopathic Pulmonary Fibrosis (IPF)

IPF is the most common form of interstitial lung disease in humans, a group of disorders most of which cause progressive scarring of lung tissue. Asbestosis and rheumatoid arthritis belong to this group. IPF in humans has been identified as an adverse effect of chemotherapy with bleomycin. The symptoms of the disease in patients are vague, including dyspnea, dry cough, and decreased exercise tolerance [164]. Typical histopathologic findings are fibrotic scarring, fibrotic remodeling, hyperplasia of AT II cells, low levels of inflammation, and thickening of the alveolar septa. Typically, heterogeneity is seen with normal lung tissue and fibrotic areas. In addition to the standard inducer, bleomycin, numerous other chemicals can cause IPF.

#### 8.5.1. Rodents

The most commonly used species is the mouse, and bleomycin, either by intratracheal, oropharyngeal, intraperitoneal, or intravenous administration, is the routinely used agent to induce the fibrotic lesions. Bleomycin hydrolase levels determine the severity of the response to bleomycin administration in mice [164]. Mice recover quickly from a single injury, but with repeated administrations, fibrosis was evident even when bleomycin administration was discontinued. The first phase manifests as acute inflammation with alveolar epithelial cell injury, inflammatory cell infiltration, and the release of inflammatory cytokines. In the subacute stage, extracellular matrix deposition and fibrotic lesions develop through the proliferation of fibroblasts and the expression of pro-fibrotic cytokines [165]. The lungs of the animals do not show the fibroblastic foci, hyperplastic epithelium, and temporal heterogeneity of human lesions. Strain differences may be pronounced, with C57BL/6 showing marked lung fibrosis and BALB/c mice being more resistant due to differential cytokine expression [83]. The response is also age- and sex-dependent, with greater fibrosis in aged males than in young females [166]. The location of the lesions depends on the route of administration. After tail vein injection, fibrosis was concentrated under the pleura and evenly distributed in the interstitial space of the lung, while after intratracheal administration, lesions were concentrated around the trachea and unevenly distributed in different lobes of the lung [84]. Intraperitoneal injection leads to fibrosis mainly concentrated under the pleura and is more similar to the pathological distribution of clinical IPF than intratracheal administration. In addition to bleomycin, a large number of other agents have been used to induce IPF in mice. Instillation of silica produces a more durable response because clearance of the particles takes longer [164]. The development of lesions, however, takes about 16 weeks, and lung heterogeneity is absent. Strain specificity is similar to induction with bleomycin. Fluorescein isothiocyanate (FITC) binds to proteins and acts as an allergen. The use has the advantage that lesions are visible in fluorescence microscopy [167]. Histopathological changes in the lung are homogeneous and dependent on the batch of FITC, which leads to problems with standardization. Irradiation as an inducer of fibrosis damages AT II cells, leading to mononuclear cell activation and combined TNF-α and TGF-β production. The extent of homogeneous fibrosis and its duration are strain-dependent. While C57Bl/6 mice are sensitive, C3H/HeJ and BCH/J mice are resistant to irradiation [166]. Problems in limiting lung fibrosis and the concomitant development of PH are negative aspects of radiation treatment. Intraperitoneal sensitization and intravenous challenge with *S. mansoni* lead to the formation of granulomas composed of lymphocytes, eosinophils, and alternatively activated macrophages, ultimately leading to IPF [168]. FITC, *S. mansoni* eggs, and irradiation have the advantage that the fibrosis is progressive, whereas bleomycin, monocrotaline, ozone, and PMA treatment only produce the acute disease [169].

IPF symptoms can also be generated by adenovirus injection [170] and transgenic models using doxycycline-regulated transgene expression. Surfactant protein C is used for AT II cells, Clara cell-10 for bronchial epithelial cells, and collagen I for fibroblasts as promoters for TGF-β overexpression. Overexpression of other cytokines (TNF-α, TGF-α, and IL-1β) by transgenic expression also leads to fibrosis. Targeted AT II cell injury is achieved by expression of the diphtheria toxin receptor under the control of a type AT II promoter [171]. Subsequent administration of diphtheria toxin selectively damages AT II cells, resulting in AT II cell hyperplasia, interstitial thickening, and collagen deposition. Spontaneous development of IPF in mice is achieved by transgenic deletion of genes for receptor of advanced glycation end products (RAGE) or for relaxin [166]. Knockout of neutrophil elastase, MMP-7, MMP-9, monocyte chemotactic protein 1, macrophage colony-stimulating factor, 5-lipoxygenase, leukotriene C4 synthase, and aortic carboxypeptidase-like protein (ACLP) protects against fibrosis and promotes fibrotic changes [97]. Deficiency of tissue and urokinase-type plasminogen activators, IL-10, cyclooxygenase 2, surfactant protein C, and phosphatase and tensin homolog (PTEN) haploinsufficiency worsens pulmonary fibrosis. Infection of aged mice with murine herpesvirus-68 also leads to fibrosis. Further, it is also possible to induce IPF by injecting fibroblasts derived from IPF patients into non-obese, severe combined immunodeficiency NOD/SCID/beige mice. The phenotype develops within 30–35 days and can be followed for months.

Instillation of monocrotaline into rats causes not only remodeling of the pulmonary vasculature and PH but also thickening of the alveolar septa, accumulation of inflammatory cells, collagen accumulation, and thickening of the pulmonary arteries [169]. The hepatotoxic agent paraquat also causes fibrosis, but not only in the lungs but also in other organs [172]. Exposure to ozone (O_3_) and nitric oxide (NO_2_) takes 3 weeks to induce lung fibrosis in rats, but the resulting pattern with centriacinar lesions is different from the peripheral pattern of human IPF [173]. Exposure must be continued to maintain the lesions. Acid instillation into the trachea of rodents resulting in acute lung injury followed by fibrosis and lung contusion-induced fibrosis in rats are not commonly used methods to mimic IPF.

Guinea pigs are used to study cough, which affects approximately 80% of IPF patients. Cough sensitivity to capsaicin and allyl isothiocyanate is increased in bleomycin-treated animals [174]. They also show evidence of lung remodeling.

#### 8.5.2. Other Small Laboratory Animals

Phorbol myristate acetate (PMA) as a leukocyte activator induces hemorrhagic pneumonitis that develops after continuous exposure to lung fibrosis in rabbits, which are in general uncommon models of IPF [169].

#### 8.5.3. Larger Animals

Bleomycin has also been used in larger animals and has induced lung fibrosis in non-human primates, dogs, and sheep [175,176,177]. Natural fibrotic disorders are seen in dogs (West Highland Terriers) [82]. The lungs show multifocal areas of subpleural peribronchioloar fibrosis, alveolar cell changes, and ground-glass opacities and are highly similar to human IPF. Dogs with natural fibrosis are nevertheless not suitable as animal model because the prevalence is low and the pathogenic mechanism is unclear.

### 8.6. Tuberculosis (Tbc)

Tbc is a bacterial infection caused by Mycobacterium tuberculosis. In addition to feeling ill and weak, weight loss, fever, and night sweats, pulmonary Tbc includes coughing, chest pain, and coughing up blood. Necrotizing granulomas, associated pneumonia, fibrosis, and dystrophic calcification are found in the lungs [178]. The infection includes the initial macrophage response, the growth phase, the immune control phase, and the lung cavitation phase. Upon inhalation of the infected aerosol, alveolar macrophages (AMs), dendritic cells, and neutrophils of the lung ingest *M. tuberculosis*. These cells migrate to the lymph nodes and induce the inflammatory response. The bacilli are phagocytosed and eliminated by activated phagocytes, but they can grow logarithmically in non-activated AM. The adaptive response/delayed-type hypersensitivity is activated within 2–10 weeks after infection. In this process, AMs harboring more than a few bacilli are killed. The combination of both immune responses leads to the formation of a mature granuloma. The granuloma typically consists of a compact core of epithelioid macrophages, dead cells, and free *M. tuberculosis*, surrounded by other immune cells such as T lymphocytes, B lymphocytes, neutrophils, dendritic cells, and giant cells (a multinucleated fusion of immune cells). They are called caseating granulomas. After liquefaction of the center and drainage through the airways, a cavern is formed. Depending on the patient’s immunity, the disease may remain dormant (latent), causing no clinical symptoms or disease, or active Tbc, in which the infection spreads to the lungs, may occur [179]. People react very differently to Tbc; about 5% of infected people would clear all pathogens, 5–10% would develop active disease, and 90% would become latent TB infection [180]. It is possible that granulomas are beneficial to the bacilli, but animal studies cannot clarify this because the structure of the granuloma is different and often different strains of mycoplasma are used.

The ideal model has to show the innate and acquired immune response with infection of phagocytes, initial control of growth (latent phase), reactivation, and finally death [181]. In addition to screening drugs against Tbc, researchers aim to develop a more effective vaccine than BCG (live attenuated *M. bovis* Bacille Calmette-Guérin). This seems to be necessary because BCG was reported to prevent only 5% of all potentially vaccine-preventable Tbc deaths [182].

#### 8.6.1. Rodents

Mice infected with *M. tuberculosis* are the most commonly used animal models. The manifestation of the disease differs from the human disease in that there is inflammation rather than the formation of caseating granulomas, and tissue destruction continues until the animal dies. Nevertheless, mouse models have been useful to identify the prominent role of T cells in containing the infection [181]. Immunodeficient mice (nude mice, SCID mice, thymectomized mice, IFN-γ^−/−^, CD4^−/−^, IL12^−/−^, TNF-α^−/−^, TLR2^−/−^, and immunosenescent mice) lack resistance to *M. tuberculosis*, and important information about the role of IFN-γ and T cells in Tbc was obtained in knockout mice [183]. IFN-γ^−/−^ mice can be used for first-line screening [184]. The latent stage of Tbc can be simulated by inoculation with *M. tuberculosis*, treatment with tuberculostatic drugs, and reactivation with steroid treatment [185].

The formation of granulomas in rats depends on the rat strain and the inoculation dose used. American cotton rats produce necrotic granulomas after pulmonary infection, in contrast to Wistar and Lewis rats, which did not show necrotic lesions [20]. Because of the different manifestations (caseating and non-caseating granulomas, abscess formation, fibrous adhesions) of the disease, this species is important in the study of Tbc.

Guinea pigs were the first models used by Robert Koch to test his postulates on infectious agents. They are also the standard models in the industry to proceed with clinical trials [186]. Infection in animals is followed by a stationary phase with hematogenous dissemination to other organs and reseeding in the lungs. In the important screening of vaccines, the problem arises that all candidates are compared to the effect of BCG and differently acting, potentially active against granuloma formation, are classified as inactive [33]. The main limitation of the drug screening model is the absence of cavitation.

#### 8.6.2. Other Small Laboratory Animals

Rabbit lungs show the same stages seen in humans, making them good models for human Tbc. However, they are not very susceptible to *M. tuberculosis* [181]. Granulomas containing *M. tuberculosis* may eventually regress and heal. *M. bovis* must be used instead, with the problem that *M. bovis* affects the intestines more than the lungs, and the bacilli are shed in the urine. Subspecies of New Zealand White rabbits have different susceptibilities to different strains of *M. bovis*. Reactivation of Tbc is caused by immunosuppression by corticoid administration [187].

#### 8.6.3. Larger Animals

Non-human primates recapitulate the human pathology of Tbc and show *M. tuberculosis* strain-specific infection. The major advantage is that the latent phase of infection can be mimicked. The extent to which the models show the natural course of human infection depends on the way the bacilli are administered [188].

Dogs can also be infected with *M. tuberculosis* and develop a disease similar to that of humans [189]. However, dogs are not used to study the mechanism or efficacy of drugs.

Minipigs develop symptoms similar to humans and are considered useful models for understanding latent Tbc infection [190]. Cattle, not a common animal model in respiratory research, can be used for vaccine testing because it is the natural host of *M. bovis* [20].

### 8.7. Non-Mammalian Models

With the enforcement of the 3Rs by the European Commission and many regulatory agencies around the world, non-vertebrate models have gained attention. It is not possible to study lung effects directly in the zebrafish (*Danio rerio*) and fruit fly (*Drosophila melanogaster*) because they do not have the relevant structures, but specific questions can be studied in these models. The main advantage of these models is that knockout variants can be easily generated to determine the effect of a specific gene.

#### 8.7.1. Zebrafish

**Figure 4:**
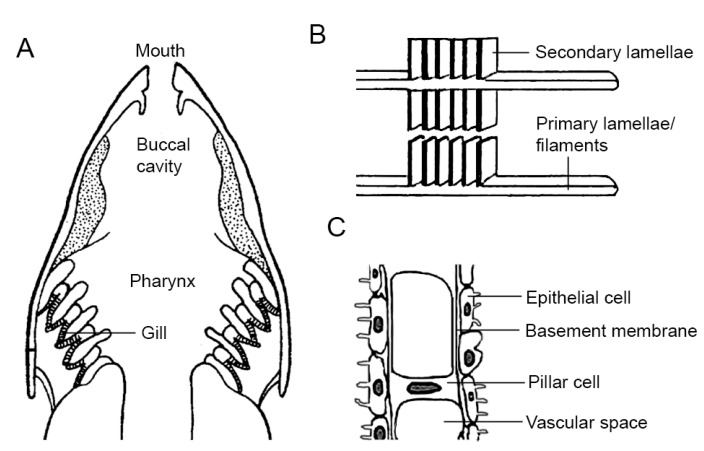
Zebrafish respiratory system. Water enters the buccal cavity through the mouth and exits the pharynx through the gills (**A**). The gills are composed of primary filaments and perpendicularly arranged secondary lamellae (**B**) to increase the surface area. The secondary lamellae consist of pillar cells and vascular space (**C**). Epithelial cells with microvilli are located on a basement membrane.

The gills are located in the pharynx, and each gill is supported by a gill arch-a bony structure that is oriented vertically on the side of the fish (Figure 4A). Filaments extend horizontally from the gill arches [191]. Each gill filament produces many primary lamellae/filaments, and the primary lamellae branch into tiny secondary lamellae (Figure 4B). The primary epithelium consists of four cell types: pavement cells, mucous or goblet cells, mitochondria-rich cells, and basal cells [192]. The secondary lamellae run parallel to the water flow and are covered by an epithelial layer on each side. The epithelial layers consist of 90% of so-called pavement cells resting on a basement membrane [193]. Their surface is enlarged by microvilli and microplicae, which help to anchor mucus. Mitochondrion-rich cells represent a minority of ~10% of the epithelial cells covering the secondary lamellae. Pillar cells provide structural support to the secondary lamellae. They have endothelial and smooth muscle cell functions and are equipped with metabolic enzymes, intercellular junctions, large amounts of smooth muscle myosin, actin, and receptors for vasoactive agents [194]. The spaces around the pillar cells and between the two epithelial layers are perfused with blood, which flows as a sheet rather than through vessels per se (Figure 4C). There are also neuroepithelial cells and a gill-associated lymphoid tissue.

Zebrafish are useful for assessing immune processes and mucus production that occur in the primary lamellae. Mucosal inflammation in the gills was induced by exposure to CS in a manner similar to asthma and COPD in the human lung [195]. In another study, gill fibrosis was observed after exposure to polyhexamethylene guanidine phosphate and irradiation [196,197].

CFTR mutations do not induce the CF phenotype, but the role of pulmonary ionocytes can be studied in zebrafish because these cells are present in their gills [198]. Pulmonary ionocytes are a rare cell type in the mammalian lung that co-express FOXI1, multiple subunits of the vacuolar-type H+-ATPase (V-ATPase), and CFTR [199].

Zebrafish are also used to study the effects of cytokines on Tbc. *M. marinum* causes granulomas that resemble human *M. tuberculosis* granulomas in the zebrafish model [200]. Reactivation of Tbc infection occurs in a similar manner as in humans [201]. Larval and adult zebrafish can be used, and all stages of the disease (active, latent, and chronic) can be mimicked by varying the *M. marinum* strain and the vaccination dose. While immune markers and granuloma morphology in adult zebrafish (5–6 weeks after fertilization) are similar to those in mammals, larvae (0–5 days after fertilization) lack an adaptive immune system. The larval model has been used to evaluate the efficacy of TB drugs such as rifampicin, isoniazid, ethambutol, and moxifloxacin [202]. Zebrafish embryos have been successfully used to identify new antimycobacterial drugs (benzathiozinones) with reduced toxicity [203]. Important limitations of this model include the difference in infection of zebrafish with *M. marinum* and *M. tuberculosis* in humans and the absence of lungs and lymph nodes in zebrafish. In contrast to chronic obstructive lung diseases, zebrafish are not useful for studying PH because they lack a separate pulmonary circulation.

#### 8.7.2. Fruit Fly

**Figure 5:**
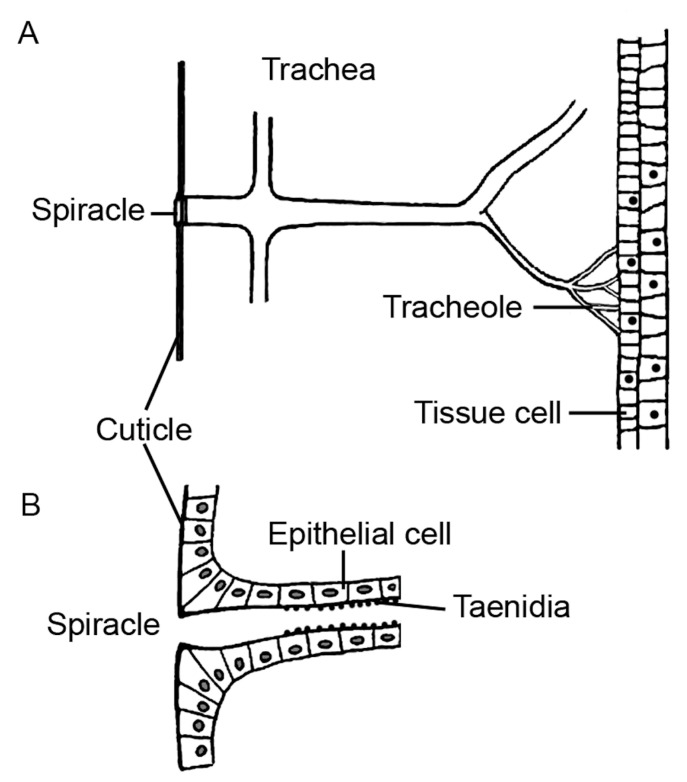
Tracheal system of the fruit fly. Air enters the insect through spiracles in the cuticle (**A**). The tracheal system forms a network of thinner branches, called tracheoles, that guide air into the tissue. The trachea is covered by epithelial cells with taenidia for stabilization (**B**).

Fruit flies use the tracheal system to exchange gases. Air enters the insect’s body through closable valves in the cuticle called spiracles (Figure 5). Airflow is regulated by small muscles that close the spiracle by contraction or open it by relaxation. There is a longitudinal tracheal trunk and a branching network of tracheal tubes that subdivide into smaller and smaller diameters. The smallest tubes are called tracheoles. They lie within specific tissue cells and sit in a pool of fluid that provides a thin, moist interface for gas exchange between atmospheric air and a living cell [204,205]. The epithelium of the trachea consists of only one type of cell (Figure 5B). To prevent its collapse, a thin wire of cuticle (the taenidia) spirals through the membranous wall [206].

The fruit fly has only one innate immune system but can use two pathogen recognition pathways: the Toll-like receptor and the immune deficiency (IMD) pathway [207]. In the tracheal epithelium, only the IMD pathway is active, and antimicrobial peptides are secreted when pathogens penetrate the epithelial barrier. Activation of the IMD pathway leads to thickening of the airway epithelium with metaplastic and hyperplastic transformations of airway epithelial cells. Instead of mucus, a protein homologous to the peritrophins, a family of chitin-binding proteins from the peritrophic matrix of insects, has been found [208].

Flies are good models for studying asthma [209]. Intuitively, one would think that they are not suitable because they lack the adaptive immune system, especially T cells and IgE. However, the identification of the important role of airway epithelial cells, the fact that asthma-related genes belong mainly to the innate immune system, and the expression of orthologs of these genes in the fly airways changed this perception. Furthermore, airway infection in flies regulates the same genes as asthma in humans. The lack of an adaptive immune system becomes an advantage because there is no need to differentiate between innate and adaptive responses. Tracheae of fruit flies have been used as a screening system for drugs in the treatment of COPD [205]. Morphology, development of the tracheal system, inflammation, generation of reactive oxygen species, mobility, and lifespan can be used as readouts. The short lifespan of the flies allows for high-throughput testing. Identification of respiratory problems is easy. While healthy flies are buried in their food, flies with respiratory problems crawl to the surface. Overexpression of miR-263a, a microRNA that negatively regulates the number of transcripts encoding the α and β subunits of ENaC, induced the CF phenotype in the gastrointestinal tract, but CF symptoms can also be seen in the airways [210]. The existing models of CFTR mutations can only mimic the effect of the gene in the gastrointestinal tract [132]. Infection of macrophages by *M. marinum* can be studied when the bacilli are injected into the abdomen of adult fruit flies [211]. The lack of a separate pulmonary circulation prevents the study of PH in *Drosophila* melanogaster. Signaling pathways for fibrosis are present in the fruit fly, but models for IPF have not been established.

## 9. General Aspects of Inhalation Testing in Animal Models

**Figure 6:**
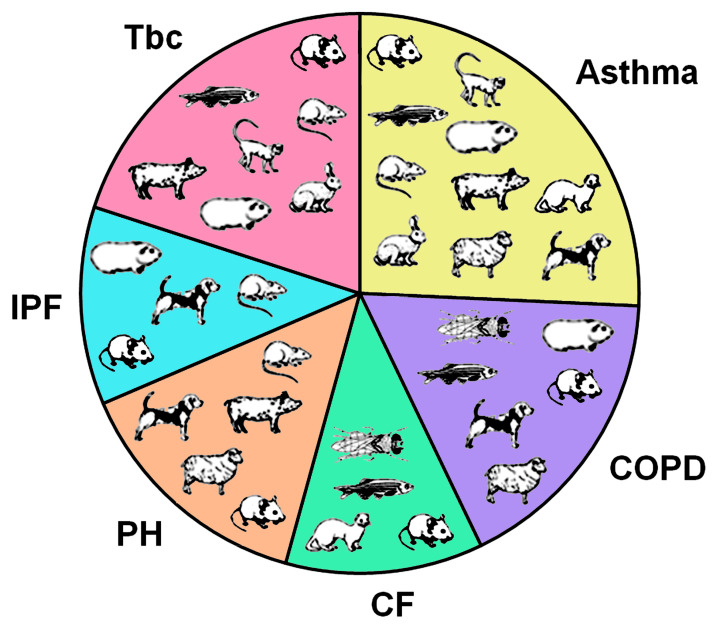
Mammalian and invertebrate models that can be used to study specific parameters of the chronic respiratory diseases.

Rarely can an animal model mimic all aspects of human disease, and the combinations of various animal models may be advantageous. Animal models that may provide relevant aspects of the disease are shown in Figure 6. Other relevant factors are ethics, cost, and practical issues (ease of handling, availability of detection reagents, use of aerosols, sample collection, and possibility of non-invasive analysis). Obviously, ethics and cost are the highest for large mammals. Procedures may be more difficult to perform in small animals (e.g., mice) and in species that bite when frightened (e.g., ferrets). Advantages and disadvantages for the use of different species as models for chronic respiratory diseases are summarized in Table 3.

Targeting the aerosol in the respiratory tract is the first challenge. Nose-only devices are suitable for rodents, and helmets, facemasks, or oropharyngeal tubes are possible for non-rodent species [212]. In general, aerosol administration and sampling become easier as the size of the animal increases. Human breathing, including breath hold, can be simulated in dogs under mechanical ventilation. Intratracheal administration delivers the dose directly to the lung. Since the upper airways and the protective mechanisms are bypassed in this procedure, effects on the lung periphery may be overestimated.

Repeated sampling of blood, biopsies, and BAL and the use of non-invasive techniques are advantages of larger animal models. The forced oscillation technique (FOT) or wedge bronchoscope technique is possible for dogs and sheep [213]. FOT involves applying low-frequency pressure oscillations to the airway opening during normal breathing [214], while the wedge bronchoscope technique is performed by passing a constant airflow into a lung segment through a bronchoscope wedged in an airway of interest and measuring pressure changes at the bronchoscope tip [126]. Both techniques provide information about airway obstruction in asthma and are used to assess airway obstruction and bronchodilator response in pediatric asthma [215].

In general, mice are considered the best species for mechanistic studies because of the availability of various transgenic and knockout strains [93]. Dose extrapolation is complicated by the large size difference between humans and mice. It is also important to note that mice recover quickly from injury, making it difficult to generate chronic models. Dosing in rats is easier, but transgenic and knockout strains are not available. The potency of sensitization and allergen responses can vary over time and with animal sources and housing. Guinea pigs can cough like larger species and have similar lung responses to allergens as humans. Their lung anatomy is also more similar to that of humans than to that of mice. They have similar limitations to rats because their allergen responses can also vary, and transgenic and knockout strains are not available. Ferrets can provide insight into the effects of glandular hyperplasia in asthma but are otherwise less commonly used in chronic respiratory disease. In rabbits, drug effects on various aspects of asthma can be studied when the animals are sensitized shortly after birth. Non-human primates (marmosets, cynomolgus, and rhesus monkeys) recapitulate the full spectrum of outcomes of Tbc infection seen in humans, but are expensive and slow, raise ethical concerns, and have limited validation data. Sheep and dogs are particularly well-suited for detailed studies of the respiratory system. These species are outbred, which introduces a source of variation. Pigs are important for training in medical procedures, but their use as disease models is less prominent. Despite the lack of a lung, zebrafish and fruit flies can be used to study the obstructive lung diseases asthma, COPD, and CF. Zebrafish can also be used for antimycobacterial drug screening.

### Induced Models versus Gene Editing Models

When creating disease models, the first idea is to use clinically relevant inducers. This is effective for asthma models with HMD, bleomycin for IPF, CS for COPD, and hypoxia for PH. The fact that the phenotype differs from the human disease is mainly due to physiological differences between species. Typically, the mucus effects of asthma cannot be studied in rodents because these species produce little mucus.

It should also be noted that the same inducer can be used to induce different diseases. CS can induce not only inflammation and emphysema, but also small airway remodeling and PH in mouse, rat and guinea pig models [216]. A similar effect is seen with bleomycin treatment, which can induce COPD, IPF, and PH. This is not unexpected as COPD and IPF are associated with a high incidence of PH in humans [217].

Genetically engineered animal models also have limitations. Strain and sex may influence the biological effect; the observed effects may be due to disruption of a gene other than the targeted one due to random integration; off-target effects may occur from the use of doxycycline in bone and embryonic development. Prolonged contact with Cre-recombinase may induce cytotoxicity [80].

## 10. Conclusions

In vitro models have improved dramatically over the past decade with the development of microfluidic systems, artificial tissues, and the incorporation of mechanical forces. Nevertheless, a complete replacement for animal testing does not seem realistic at this time.

Rather than a complete replacement, it is promising to obtain the maximum amount of information from controlled animal studies. Choosing an animal model based solely on practical benefits and costs rather than on the expression of the desired phenotype does not seem ideal. Large animals offer the possibility of using imaging and invasive examination (bronchoscopy), and different parts of the lung can be treated differently. Transgenic animals have an established role in medical research. Cryopreservation of oocytes and sperm is a way to save animals in breeding when a transgenic strain is not needed for a certain period of time. The mandatory reporting of animal experiments according to the Animal Research: Reporting of In Vivo Experiments (ARRIVE) guideline could be one way to ensure that only high-quality studies are published [218].

It is likely thatthe training of human surgical procedures will continue to rely on the use of animals, mainly pigs. The use of more species, but fewer in number, to address specific parameters in respiratory disease, in combination with in silico and cellular models, will improve the efficacy of drugs in clinical trials. Artificial intelligence (AI) tools and advanced in vitro models will reduce the number of animals used in toxicity testing to a small number of regulatory in vivo toxicity tests for first-in-human studies. Invertebrate testing is likely to play a greater role in the future. It is possible that other transgenic animals will replace mice in mechanistic studies. The potential of AI to further reduce or even replace animal testing is not yet clear [219].

## Figures and Tables

**Table 1:** Overview of species differences in tracheobronchial airway organization compared to humans (animal order according to size).

Parameter	Human	Mouse	Rat	Guinea Pig	Rabbit	Ferret	Monkey	Dog	Sheep	Pig
**Breathing**	Mouth and nose	Obligatory nose	Obligatory nose	Obligatory nose	Obligatory nose	Mouth and nose	Mouth and nose	Mouth and nose	Mouth breathing possible	Mouth and nose
**Cough reflex**	present	absent	absent	present	present	absent	present	present	present	present
**Lung architecture**	5 lobes, 2 left, 3 right	5 lobes, 1 left, 4 right	5 lobes, 1 left, 4 right	7 lobes, 3 left, 4 right	7 lobes, 3 left, 4 right	6 lobes: 2 left, 4 right	6 lobes: 2 left, 4 right	6 lobes: 2 left, 4 right	6 lobes: 2 left, 4 right	6 lobes: 2 left, 4 right
**Branching**	Dichotomous	Monopodial	Monopodial	Monopodial	Monopodial	Monopodial	Monopodial	Monopodial; dichotomous	Dichotomous	Monopodial
**Branchings to alveolarized bronchiole**	17–21	13–17	8–25 or 13–32, depending on lobe	14	32–36	>6	13–17	15–22	7–13	21–23
**Submucosal glands**	Trachea, bronchi	Trachea (1/3)	Trachea (1/3)	Trachea (1/3)	Absent	Trachea, bronchi	Trachea, bronchi	Trachea, bronchi	Trachea, bronchi	Trachea, bronchi
**Other**		Chest wall less stiff	Chest wall less stiff	Chest wall less stiff	Chest wall less stiff		No interlobular septa	No interlobular septa	Prominent interlobar septa	Prominent interlobar septa
**References**	[25,26]	[25,26]	[25,26,27]	[25,26,28]	[25,26,29,30]	[31,32]	[25,26,33]	[25,34,35]	[25,36,37]	[25,36,38]

**Table 2:** Interspecies differences in the cellular composition of the airways from trachea to bronchi generation 2–6.

Parameter	Human	Mouse	Rat	Guinea Pig	Rabbit	Ferret	Monkey	Dog	Sheep	Pig
Thickness of epithelial layer (μm)	100–50	14–11	24–13	11	21–29	17–20	30–20	33	59–32	50–30
Cells/mm BM	303 ± 20	215	126–116	307	194–114	n.a.	181 ± 51	n.a.	285–284	303
Ciliated cells (%)	49	39	35–53	32	43–49	Cells/mm: 80–20	33	n.a.	48–39	43
Mucous goblet cells (%)	9	<1	<1	5	1	Cells/mm: 20–60	17	9.6	4–8	3
Serous cells (%)	n.a.	21	0	0	0	n.a.	<1	n.a.	0	0
Club (Clara) cells (%)	n.a.	49	0	n.a.	22–41	n.a.	<1	n.a.	n.a.	n.a.
Basal cells (%)	33	10	27–14	34	27–49	n.a.	42	n.a.	18–19	31
Ref.	[53]	[54]	[53]	[55]	[53,56]	[32]	[57]	[58]	[53]	[55]

Abbreviation: BM: basal membrane; n.a.: not available.

**Table 3:** Advantages and disadvantages of mammalian species and invertebrates for the study of chronic respiratory diseases.

Species	Advantages	Disadvantages
Mouse	Low costs, many providers, short breeding time, easy handling, comparably low ethical considerations, most test reagents available, transgenic animals, vast literature data, numerous inbred strains, good for mechanistic studies	Small size (aerosol delivery difficult, sample volumes small), short life span, different lung structure, obligatory nose breathers, low mucus production, limited airway musculature, no chronic models, strain-specific responses
Rat	Larger lung surface than other rodents, good for pharmacodynamic and toxicological testing	Strain-specific responses, obligatory nose breathers, higher mucociliary clearance, different airway macrostructure and epithelial composition
Guinea pig	Best models for inflammation and AHR (asthma, Tbc)	Shortage of inbred strains, axon reflex, difficult blood collection because of thick skin and lack of tail, few reagents available.
Rabbit	Procedures (tracheotomy) like for large animals, good availability, easy handling, longer observation times	Difficult intubation, differences in airway architecture and epithelial composition
Ferret	Comparable respiratory tract, ideal models for COPD, genetically modified animals available	Limited availability, complex husbandry, not fully annotated genome, few inbred strains, handling difficult (biting), reagents not easily available
Non-human primates	Genetic and morphological similarity to humans, reagents available due to cross-reactivity with human	Ethical problems, high costs, skilled handling and specialized equipment necessary
Dog	Easy intubation due to large mouth opening, greater number of alveoli than rodents, broad spectrum of breeds, use of human inhalers possible	Ethical problems, larger airways make identification of constriction difficult, difference due to outbred strains
Sheep	Similar mucus composition to humans, use of human devices possible, lungs can be treated separately, serve as surrogate models for surfactant dysfunction	High costs, intense labor, vomiting possible upon intubation
Pig	Genetic homology, body weight, metabolism, organ structure similar to humans, human devices can be used, long observation time, genetically modified animals available	Differences in pharyngeal anatomy, handling more difficult, higher costs, laryngospasm possible upon intubation
Zebrafish	Costs low, large number of eggs, transgenic animals	No lung
Fruit fly	High-throughput testing possible, simple protocols	Low conserved homology with human genome, no lung

## Data Availability

Data included in the work are available in the referenced literature.
